# Integration of gut microbiota, Stroke Dysbiosis Index, and inflammatory biomarkers in assessing prognosis of acute ischemic stroke

**DOI:** 10.3389/fmed.2025.1707253

**Published:** 2026-02-02

**Authors:** Weny Rinawati, Aryati Aryati, Abdulloh Machin, Stefan Kiechl, Gregor Broessner

**Affiliations:** 1 Doctoral Program in Medical Science, Faculty of Medicine, Universitas Airlangga, Surabaya, Indonesia; 2 Department of Supporting Medicine, National Brain Center Hospital Mahar Mardjono, Jakarta, Indonesia; 3 Laboratory and Blood Bank Unit, National Brain Center Hospital Mahar Mardjono, Jakarta, Indonesia; 4 Department of Clinical Pathology, Faculty of Medicine, Universitas Airlangga, Surabaya, Indonesia; 5 Dr. Soetomo General Academic Hospital, Surabaya, Indonesia; 6 Department of Neurology, Faculty of Medicine, Universitas Airlangga, Surabaya, Indonesia; 7 Airlangga University Hospital, Surabaya, Indonesia; 8 Department of Neurology, Medical University of Innsbruck, Innsbruck, Austria; 9 VASCage - Center for Clinical Stroke Research, Innsbruck, Austria

**Keywords:** acute ischemic stroke, gut microbiota, dysbiosis, inflammatory biomarkers, prognosis

## Abstract

**Background:**

Acute ischemic stroke (AIS) is often complicated by systemic infections that worsen prognosis. Emerging evidence suggests gut microbiota dysbiosis, inflammatory biomarkers, and gut–barrier dysfunction play pivotal roles in post-stroke outcomes. This study aimed to integrate Stroke Dysbiosis Index (SDI), Microbial Dysbiosis Index (MDI), and inflammatory biomarkers to evaluate their prognostic value in AIS patients.

**Methods:**

An observational prospective cohort was conducted at a tertiary stroke center in Jakarta (September 2023–September 2024). Eighty AIS patients admitted within 24 h of onset were enrolled. Fecal samples underwent 16S rRNA sequencing for microbiota profiling and SDI/MDI calculation. Blood biomarkers (NMDAR NR2B, butyrate, TMAO, RANKL, iFABP, LPS) and platelet-to-lymphocyte ratio (PLR) were measured. Clinical severity was assessed with NIHSS. Outcomes included infection within 7 days, and complications. Statistical analyses comprised correlation, regression, and ROC curve modeling.

**Results:**

Infection occurred in 46.3% of patients, predominantly older (>60 years) and female. Infected patients showed reduced microbial diversity, enrichment of pathogenic taxa (Klebsiella, Escherichia, Salmonella), elevated SDI/MDI, and depleted SCFA producers. Biomarkers revealed increased NMDAR, TMAO, iFABP, and LPS, with reduced butyrate and RANKL (all *p* < 0.001). These markers correlated strongly with infection status. ROC analysis demonstrated promising discriminative ability in this cohort (bias-corrected AUCs > 0.80 after internal bootstrapping validation). Internal validation using 1,000 bootstrap resamples was performed to estimate optimism in AUC values and obtain bias-corrected confidence intervals.

**Conclusion:**

Gut dysbiosis, elevated dysbiosis indices, and inflammatory biomarker derangements were strongly associated with post-stroke infections and adverse prognosis. Integrating microbiota and biomarker profiles with clinical parameters may provide a robust framework for risk stratification and open avenues for microbiota-targeted therapies in AIS.

## Introduction

Acute ischemic stroke (AIS) remains one of the leading causes of disability and mortality worldwide. In the last decade, the gut–brain axis has highlighted the intestinal microbiota as a key component modulating systemic inflammation, immunity, and ultimately neurological outcomes after stroke. Dysbiosis, defined as an imbalance in microbial composition and function, is characterized by decreased diversity, depletion of beneficial microbes (e.g., short-chain fatty acids/SCFAs producers), and enrichment of opportunistic taxa, all of which contribute to inflammatory susceptibility and poor neurological prognosis (1).

A quantitative approach to assess stroke-specific dysbiosis has been introduced through the Stroke Dysbiosis Index (SDI). In AIS cohorts, SDI, derived from differences in gut taxa between patients and healthy controls, was significantly higher in stroke patients, correlated with baseline National Institutes of Health Stroke Scale (NIHSS) and discharge modified Rankin Scale (mRS), and predicted functional outcomes. Transfer of gut microbiota from patients with high SDI into mouse models exacerbated infarct volume and neurological deficits, supporting a causal role of dysbiosis in brain injury (2).

Meanwhile, the Microbial Dysbiosis Index (MDI), a generic index combining enriched and depleted taxa, has been applied across multiple diseases and shown utility in summarizing dysbiosis into a standardized metric. In AIS, integration of depleted taxa (e.g., Roseburia, SCFA producers) and enriched opportunistic taxa into MDI successfully identified the risk of stroke-associated pneumonia (SAP), where higher MDI values were associated with nearly two-fold increased SAP risk in both derivation and validation cohorts (3, 4).

Age is a critical determinant of gut microbiota composition and immune responsiveness. Biological aging is associated with decreased microbial diversity, a shift toward pro-inflammatory taxa, and “inflammaging,” which exacerbates immune dysfunction after stroke. Thus, age may modulate SDI/MDI values and their association with clinical outcomes (1).

In addition to dysbiosis indices, simple hematological biomarkers reflecting systemic inflammation—such as the platelet-to-lymphocyte ratio (PLR), have demonstrated prognostic value in AIS. Multiple observational studies and meta-analyses reported that higher PLR is associated with poorer functional outcomes, increased mortality, and higher risk of adverse events after interventions (e.g., thrombolysis). Dynamic changes in PLR before and after thrombolysis also illustrate evolving short-term risk, positioning PLR as a promising inflammatory marker that can be integrated with SDI/MDI and NIHSS for prognostic modeling (5–7).

The correlation of dysbiosis with biological biomarkers further strengthens mechanistic plausibility. In AIS patients with SAP, decreased fecal SCFA levels—crucial for maintaining gut barrier integrity and regulating immune responses—were observed alongside increased serum D-lactate, a marker of bacterial translocation, indicating “gut barrier failure.” In parallel, systemic inflammatory biomarkers such as procalcitonin (PCT) have been extensively studied for the diagnosis and prediction of SAP and may complement microbiota indices in predictive models (8).

Gut dysbiosis is also linked to initial stroke severity and mid-term recovery. Patients with poor 3-month outcomes exhibited an enrichment of pathogenic bacteria and depletion of SCFA producers at baseline. Both clinical and pre-clinical studies revealed that stroke itself rapidly induces dysbiosis (e.g., Enterobacteriaceae overgrowth), aggravating infarct development, thus reflecting a bidirectional relationship between brain and gut (8, 9).

From the perspective of clinical outcomes, SAP is the most frequent and clinically significant complication in AIS, strongly associated with increased mortality and disability. Microbiota profiles, whether individual taxa or composite indices (SDI/MDI), have improved predictive accuracy for SAP beyond clinical factors alone (e.g., NIHSS), thereby opening opportunities for personalized preventive strategies. Beyond SAP, dysbiosis and systemic inflammation may also be linked to other complications (e.g., pro-coagulant states) and mortality, emphasizing the relevance of integrating microbiota–inflammatory indicators (9).

Based on this evidence, the present study is designed to integrate SDI, MDI, and inflammatory biomarkers, particularly PLR, alongside clinical severity scores (NIHSS) to evaluate prognosis in AIS patients. The main objectives are: (1) to characterize the association of SDI/MDI with age and baseline stroke severity; (2) to examine correlations between SDI/MDI and systemic inflammatory biomarkers; and (3) to assess the predictive performance of microbiota–inflammatory–clinical parameters for infection, mortality, and post-stroke complications. This integrative approach is expected to yield a more accurate risk stratification model applicable in the acute phase of AIS and provide biological rationale for targeted preventive and therapeutic strategies (2, 4).

## Methods

### Study design and setting

This was an observational analytic study with a prospective cohort design conducted at Prof. Dr. Mahar Mardjono National Brain Center Hospital, Jakarta, Indonesia, which is a tertiary referral center for stroke care. The study was carried out between October 2023 and September 2024 after receiving approval from the Ethics Committee of the National Brain Center Hospital Prof. Dr. Mahar Mardjono, Jakarta (Approval No. DP.04.03/D.XXIII.9/132/2023, dated October 16, 2023). This study represents a predefined sub-analysis conducted on the same acute ischemic stroke (AIS) cohort as reported in our primary cohort publication.

### Study population and sample size

The study population comprised patients diagnosed with AIS by reliable clinical symptoms and/or confirmed by neuroimaging (CT scan or MRI) according to WHO-MONICA criteria. Inclusion criteria were: (1) adult patients aged ≥18 years, (2) diagnosis of AIS confirmed clinically and radiologically, and (3) ability to provide fecal and blood samples within the first 72 h of admission. Exclusion criteria included: (1) prior antibiotic, probiotic, or prebiotic use within the last 4 weeks, (2) gastrointestinal diseases that may affect microbiota composition, (3) immunocompromised state or malignancy, and (4) refusal to participate. Sample size was determined based on the formula for cohort studies with minimum power 80% and confidence interval 95%, using effect size data from previous studies on gut microbiota dysbiosis in AIS. A total of 80 patients were recruited consecutively.

*A priori* sample size calculation was conducted using G*Power 3.1 based on an anticipated medium effect size (Cohen’s *d* = 0.65) derived from previous gut–brain axis studies in stroke (2, 6), a significance level *α* = 0.05, and a desired power (1–*β*) = 0.80. The resulting minimum required sample size was 72 subjects. To compensate for potential dropouts, 80 patients were enrolled. Given the exploratory design and number of parameters included, the study may still be underpowered for multivariate analyses, and findings should be interpreted with caution.

### Variables and operational definitions

Gut microbiota profile was assessed using 16S rRNA sequencing of fecal samples. The Stroke Dysbiosis Index (SDI) was calculated based on the relative abundance of stroke-associated bacterial taxa compared with healthy reference controls. Microbial Dysbiosis Index (MDI) was calculated as the log ratio of the total abundance of taxa increased in AIS over the total abundance of taxa decreased in AIS. Stroke severity was measured by the National Institutes of Health Stroke Scale (NIHSS) on admission. Platelet-to-lymphocyte ratio (PLR) was calculated from complete blood count obtained at baseline. The Stroke Dysbiosis Index (SDI) was calculated according to the method described by Xia et al. (2), using the log ratio of stroke-associated taxa (positively weighted) to control-associated taxa (negatively weighted). The healthy reference control group for SDI derivation was obtained from previously published datasets of age- and sex-matched healthy Indonesian volunteers.

The Microbial Dysbiosis Index (MDI) was computed as:


$$\mathrm{MDI}=\log10\;[\begin{array}{l} (1+\Sigma \;\text{abundance of pathogenic taxa})/\\ \left(1+\Sigma \;\text{abundance of beneficial taxa}\right) \end{array}],$$

following the approach by Duvallet et al. (4).

Biomarkers included serum N-methyl-D-aspartate receptor antibody (NMDAR NR2B), butyrate, trimethylamine-N-oxide (TMAO), receptor activator of NF-κB ligand (RANKL), intestinal fatty acid-binding protein (iFABP), and lipopolysaccharide (LPS). Biomarkers were measured by enzyme-linked immunosorbent assay (ELISA) using standardized commercial kits.

Clinical outcomes included: (1) stroke-associated infection (pneumonia, urinary tract infection, or sepsis) within 7 days, defined by established criteria (CDC and Sepsis-3 definitions); (2) in-hospital mortality; and (3) other systemic complications (renal failure, sepsis, etc.). Functional outcome at discharge was evaluated using modified Rankin Scale (mRS).

### Data collection procedures

Fecal specimens were obtained within 72 h of admission, collected in DNA/RNA Shield fecal tubes, and stored at −80 °C until analysis. Microbiota profiling was performed using 16S rRNA sequencing on the Illumina platform. Stroke Dysbiosis Index (SDI) and Microbial Dysbiosis Index (MDI) were calculated based on microbial composition relative to healthy controls.

Venous blood specimens (4 mL) were collected in serum activator tubes, left at room temperature for 2 h, and centrifuged (3,000 rpm, 15 min). Serum was aliquoted and stored at −80 °C. Biomarkers analyzed included NMDAR NR2B, butyrate, TMAO, RANKL, iFABP, and LPS using ELISA with standard calibration curves. Platelet-to-lymphocyte ratio (PLR) was calculated from baseline complete blood count.

To control for infection status, a procalcitonin (PCT) test was performed at baseline. A second PCT test was conducted either at day 7 (for non-infected patients) or at the time of infection onset, allowing grouping into infection and non-infection categories.

### Statistical analysis

Descriptive statistics were used to summarize baseline characteristics. Continuous variables were expressed as mean ± SD or median (IQR), and categorical variables as frequencies and percentages. Comparisons between groups (e.g., patients with and without SAP, survivors vs. non-survivors) were performed using Student’s *t*-test or Mann–Whitney *U*-test for continuous variables and chi-square or Fisher’s exact test for categorical variables. Correlation analyses between SDI/MDI, PLR, NIHSS, and biomarkers were performed using Spearman’s or Pearson’s correlation coefficients as appropriate. Logistic regression was applied to identify independent predictors of clinical outcomes (infection, mortality, complications). Variables with *p* < 0.10 in univariate analysis were included in the multivariate model.

Receiver operating characteristic (ROC) curves were generated to assess the discriminative power of combined models (SDI, MDI, PLR, biomarkers, and NIHSS) in predicting outcomes. A two-tailed *p* < 0.05 was considered statistically significant. Statistical analyses were performed using SPSS version 26 (IBM Corp., Armonk, NY, United States).

To control for multiple comparisons, *p*-values from correlation and taxonomic analyses were adjusted using the Benjamini–Hochberg False Discovery Rate (FDR) correction, with adjusted *p* < 0.05 considered significant.

## Results

### Patient characteristics

A total of 80 patients with acute ischemic stroke were enrolled, comprising 37 patients (46.3%) in the infection group and 43 patients (53.7%) in the non-infection group. The mean age of the infection group was significantly higher compared with the non-infection group (67.2 ± 10.4 vs. 57.8 ± 11.6 years, *p* = 0.004). Patients above 60 years were more likely to develop infections (77.8% vs. 43.2%, *p* = 0.006). Male patients predominated overall (57.5%), with a significantly higher proportion in the non-infection group (69.8% vs. 45.9%, *p* = 0.027). Body mass index (BMI) analysis showed that obesity was common, especially among non-infection patients (65.1% vs. 45.9%, *p* = 0.029). Other cardiovascular risk factors such as hypertension, diabetes mellitus, dyslipidemia, and hyperuricemia did not differ significantly between groups. Stroke severity on admission, assessed by NIHSS, was slightly higher in the infection group but did not reach statistical significance (median NIHSS 5.0 vs. 4.0, *p* = 0.077). Dysphagia was more frequent among infection patients (29.7% vs. 11.6%, *p* = 0.041). Intensive care admission and invasive procedures such as nasogastric tube (NGT), central venous catheter, and urinary catheter placement were more frequent in the infection group, though only total parenteral nutrition (TPN) reached significance (13.5% vs. 0%, *p* = 0.013). Laboratory findings revealed that leukocytosis (>10,000/µL) was significantly more common among infection patients (62.8% vs. 32.4%, *p* = 0.006) (Tables 1–3).

**Table 1:** Characteristic patients.

	Total		Infection		Non-infection		*p*-value
	*n*	(%)	*n*	(%)	*n*	(%)	
Subjects	80	100	37	46.3	43	53.7	
Age, mean (SD)	62.1 (12.0)		67.2 (10.4)		57.8 (11.6)		0.004*
Age group, *n* (%)							
<60	33	41.3%	8	22.2	25	56.4	0.006*
≥60	47	58.7%	28	77.8	19	43.2	
Sex, *n* (%)							
Male	46	57.5	17	45.9	30	69.8	0.027*
Female	34	42.5	20	54.1	13	30.2	-
Body mass index (BMI), *n* (%)							
*Underweight* (<18.5)	1	1.3	1	2.7	-	-	0.029*
Normal (18.5–22.9)	17	21.3	12	32.4	5	11.6	
*Overweight* (23.0–24.9) 17		21.3	7	18.9	10	23.3	
Obesity (>25.0)	45	56.3	17	45.9	28	65.1	
Cardiovascular risk factors, *n* (%)							
Hypertension	49	61.3	21	56.8	28	65.1	0.296
Diabetes mellitus	35	43.8	16	43.2	19	44.2	0.556
Dyslipidemia	58	72.5	30	81.1	28	65.1	**0.089**
Hyperuricemia	15	18.8	8	21.6	7	16.3	0.372
Smoking	6	7.5	2	5.4	4	9.3	0.509
Diet, *n* (%)							
High sodium	34	42.5	17	45.9	17	39.5	0.362
High sugar	35	43.8	19	51.4	16	37.2	**0.148**
High fat	35	43.8	13	35.1	22	51.2	**0.112**
High purine	14	17.5	6	16.2	8	18.6	0.508
Low fiber	3	3.8	2	5.4	1	2.3	0.470
Score NIHSS, median (Q1–Q3)	4.5 (4.0–9.0)		5.0 (4.0–5.0)		4.0 (4.0–7.0)		0.077
Stroke severity at admission, *n* (%)							
Mild stroke	40	50.6	15	41.7	25	58.1	**0.074**
Moderate stroke	33	41.8	16	44.4	17	39.5	
Moderate–severe stroke	6	7.6	5	13.9	1	2.3	
Severe stroke	-	-	-	-	-	-	

Bold values denote statistically significant results (*p* < 0.05). “*” indicates statistical significance at *p* < 0.05.

**Table 2:** Laboratory and clinical biomarkers.

Biomarker	Infection	Non-infection	*p*
NMDAR, ng/mL	3.99 (3.28–9.20)	1.23 (0.82–2.67)	<0.001*
Butirat, ng/mL	7.59 (4.16–9.74)	13.22 (11.81–15.56)	<0.001*
TMAO, ng/mL	636.46 (569.08-691.52)	396.22 (299.13–478.63)	<0.001*
RANKL, pg/mL	4.51 (3.92–5.53)	43.92 (6.40–83.09)	<0.001*
iFABP, pg/mL	4.35 (3.43–5.57)	2.43 (1.79–3.02)	<0.001*
LPS, pg/mL	190.80 (14.01–251.93)	83.00 (63.94–122.00)	<0.001*

“*” indicates statistical significance at *p* < 0.05.

**Table 3:** Correlation between biomarkers and infection status.

Characteristics		NMDAR	Butirat	TMAO	RANKL	iFABP	LPS
Infection event	*r*	0.711	−0.632**	0.631**	−0.638**	0.681**	0.685**
	*p*	<0.001*	<0.001*	<0.001*	<0.001*	<0.001*	<0.001*

“*” indicates statistical significance at *p* < 0.05. “**” indicates results remaining significant after FDR correction.

### Inflammatory and gut-barrier biomarkers

Biomarker profiling demonstrated significant differences between infected and non-infection group. NMDAR concentrations were higher in infected patients (3.99 ng/mL vs. 1.23 ng/mL, *p* < 0.001). Butyrate levels were markedly lower in infections (7.59 ng/mL vs. 13.22 ng/mL, *p* < 0.001). TMAO concentrations were elevated in infections (636.46 μg/mL vs. 396.22 μg/mL, *p* < 0.001), while RANKL levels were lower (4.51 μg/mL vs. 43.92 μg/mL, *p* < 0.001). iFABP, a marker of gut barrier injury, was significantly higher in infected patients (4.35 ng/mL vs. 2.43 ng/mL, *p* < 0.001). Similarly, plasma LPS was elevated (190.8 ng/mL vs. 83.0 ng/mL, *p* < 0.001).

Correlation analysis confirmed strong associations between these biomarkers and infection status: NMDAR (*r* = 0.711, *p* < 0.001), iFABP (*r* = 0.681, *p* < 0.001), LPS (*r* = 0.685, *p* < 0.001), and TMAO (*r* = 0.631, *p* < 0.001) were positively correlated with infection, whereas butyrate (*r* = −0.632, *p* < 0.001) and RANKL (*r* = −0.638, *p* < 0.001) showed negative correlations.

### Gut microbiota diversity and composition

Microbiome analysis revealed significant dysbiosis in infected patients. Alpha diversity indices showed reduced richness and evenness in infections: Shannon index (*p* = 0.0229) and Pielou index (*p* = 0.0264) were significantly lower, while Simpson and Chao1 indices did not differ.

Beta diversity analysis using Bray–Curtis distances showed clear compositional separation between groups (PERMANOVA R^2^ = 0.06, *p* = 0.005), while BETADISPER confirmed that group differences were not due to dispersion effects (*p* = 0.241). PCoA and PCA biplots identified taxa driving group separation, with pathogenic species such as *Klebsiella pneumoniae*, *Escherichia coli*, and *Salmonella enterica* enriched in infections, and beneficial commensals such as *Faecalibacterium prausnitzii*, *Blautia wexlerae*, and Agathobacter rectalis enriched in non-infections.

Taxonomic profiling confirmed that Bacillota, Bacteroidota, and Pseudomonadota were dominant phyla in both groups, but the infection group was characterized by enrichment of Enterobacteriaceae and Streptococcaceae, while Lachnospiraceae and Ruminococcaceae were reduced. At the genus and species level, pathogenic enrichment included Klebsiella, Escherichia, and Salmonella, whereas SCFA producers such as Blautia, Faecalibacterium, and Agathobacter were significantly depleted.

### Dysbiosis and functional signatures

**Figure 1:**
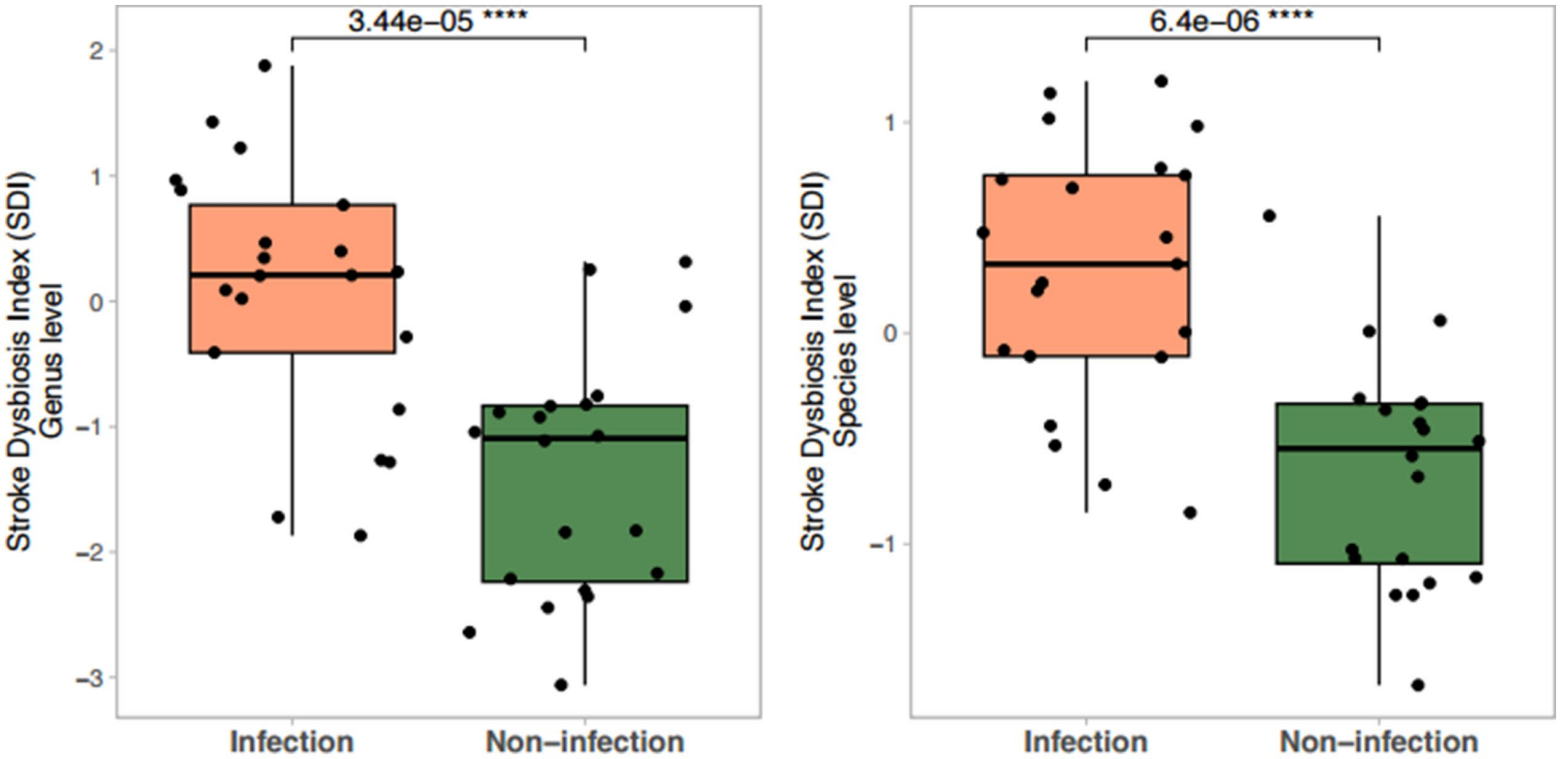
Stroke Dysbiosis Index (SDI) across groups at genus and species levels.

**Figure 2:**
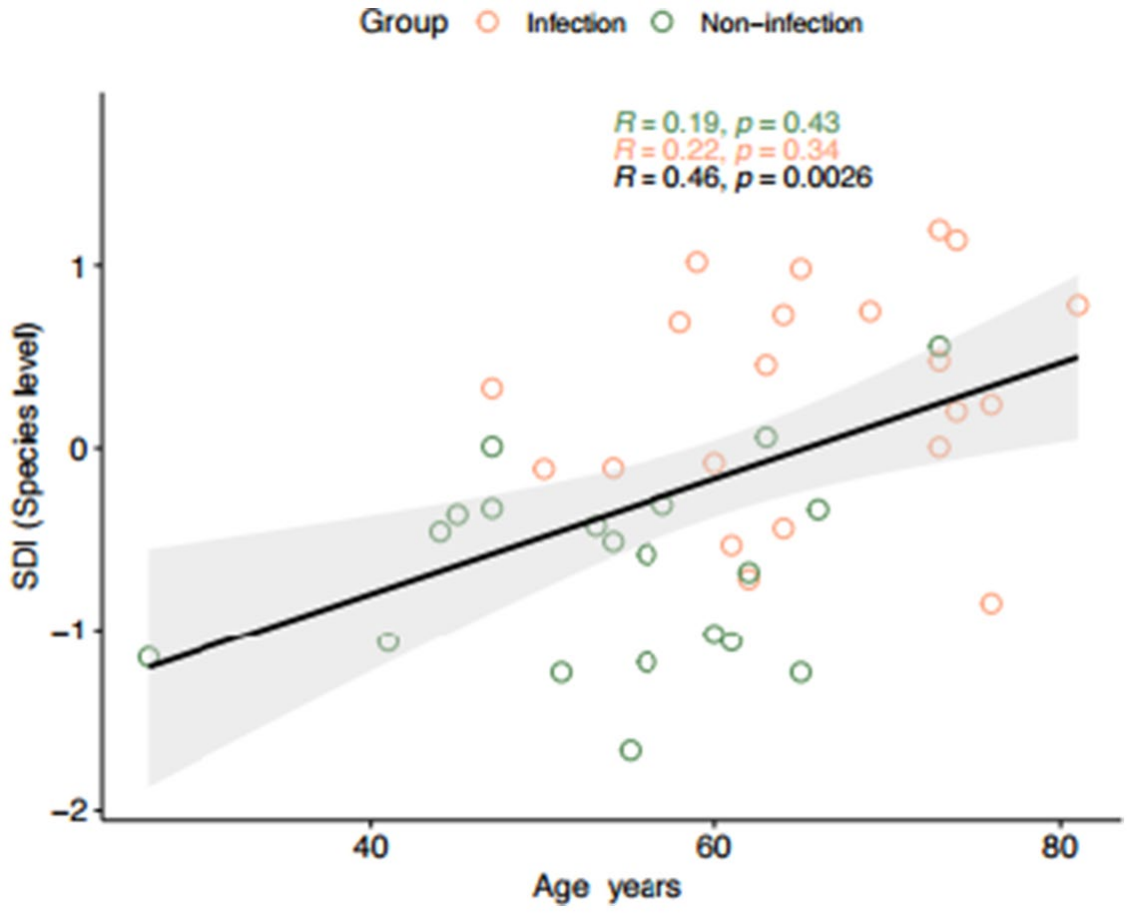
Spearman correlation between age and Stroke Dysbiosis Index (SDI) at the species level. The correlation was analyzed separately for infection (red) and non-infection (green) groups, as well as for the pooled dataset (black). In the infection group, the correlation was moderate (*R* = 0.22, *p* = 0.34), while in the non-infection group it was weak (*R* = 0.19, *p* = 0.43). A significant positive correlation was observed in the combined group (*R* = 0.46, *p* = 0.0026), indicating an age-related increase in SDI across the cohort. All other results are provided in the folder: SDI.

The Stroke Dysbiosis Index (SDI) and Microbiome Dysbiosis Index (MDI) were both significantly elevated in infection groups (*p* < 0.0001). Metabolite-associated microbial analysis revealed reduced abundance of butyrate producers and increased prevalence of TMA-producing bacteria in infected patients, consistent with higher systemic TMAO levels (Figures 1, 2).

### Microbial co-occurrence networks

Network analysis revealed a loss of microbial connectivity in infection groups. Compared with non-infected patients (281 nodes, 1821 edges), infected patients had fewer nodes (121) and edges (402), with networks dominated by pathogenic taxa and diminished cooperative interactions. These findings suggest an ecological collapse of the gut microbiome in infection states.

## Discussion

This study provides new insights into the interplay between gut microbiota composition, dysbiosis indices, and systemic inflammatory biomarkers in determining prognosis after acute ischemic stroke (AIS). We demonstrated that patients who developed infections post-stroke exhibited marked reductions in microbial diversity, enrichment of pathogenic taxa, depletion of short-chain fatty acid (SCFA)-producing bacteria, elevated Stroke Dysbiosis Index (SDI) and Microbial Dysbiosis Index (MDI) scores, as well as significant alterations in inflammatory and gut-barrier biomarkers including NMDAR, TMAO, RANKL, butyrate, iFABP, and LPS. Collectively, these findings highlight a multifactorial pathogenic model in which microbiome imbalance, metabolic derangements, and systemic inflammation converge to shape stroke outcomes.

The observed correlation between age and SDI (*r* = 0.46, *p* = 0.0026) in the combined cohort likely reflects between-group differences (infected vs. non-infected) rather than a uniform linear relationship across all patients. Within-group analyses did not show significance, suggesting that age may act as a confounding or effect-modifying factor rather than a direct determinant of dysbiosis. This nuance highlights the need for larger cohorts to disentangle age effects from infection-related microbial alterations.

### Gut dysbiosis and microbial diversity

Our data revealed that alpha diversity indices (Shannon and Pielou) were significantly lower in infected patients, confirming that reduced microbial richness and evenness are hallmarks of post-stroke dysbiosis. This aligns with previous studies reporting diminished diversity in AIS patients, which weakens resilience against pathogenic invasion and predisposes to systemic complications (10). Beta diversity analyses showed clear compositional separation between infected and non-infection group, with enrichment of *Klebsiella pneumoniae*, *Escherichia coli*, and *Salmonella enterica*, while beneficial taxa such as *Faecalibacterium prausnitzii* and *Blautia wexlerae* were depleted. Such patterns mirror earlier findings that Enterobacteriaceae expansion and SCFA-producer depletion are consistent microbial signatures of poor stroke outcomes (3, 11–14).

### Functional dysbiosis and stroke prognosis

The significant elevation of SDI and MDI in the infection group underscores the clinical utility of composite dysbiosis indices. Previous work has validated SDI as an independent predictor of stroke severity and early functional outcomes (2). Our findings extend this evidence by linking elevated SDI/MDI with systemic infections and biomarker abnormalities, strengthening the case for using microbiota-derived indices in clinical risk stratification. This metabolic imbalance corresponded to reduced systemic butyrate levels and elevated TMAO, consistent with earlier studies showing that depletion of SCFA producers impairs neuroprotection, while TMAO promotes vascular inflammation and worsens prognosis (6, 15–17).

### Inflammatory and gut-barrier biomarkers

Our results highlight profound disturbances in systemic inflammatory and gut-barrier markers. Elevated iFABP and LPS levels in infected patients indicate compromised intestinal integrity and endotoxemia, mechanisms previously linked to neuroinflammation and secondary brain injury (1, 9). The strong correlation between LPS and TMAO with infection risk further suggests that gut-derived molecules actively contribute to systemic inflammation and poor outcomes. Conversely, butyrate depletion and reduced RANKL levels reflect impaired mucosal immunity and dysregulated host–microbe signaling, both critical to maintaining homeostasis. These findings resonate with mechanistic studies showing that SCFAs, particularly acetate and butyrate, attenuate neuroinflammation and improve recovery after stroke (6).

### Microbial networks and ecological collapse

Network analysis demonstrated reduced connectivity in infected patients, with fewer nodes and edges dominated by pathogenic taxa. This ecological collapse is consistent with prior reports that stroke-induced dysbiosis disrupts microbial cooperation, favoring opportunistic overgrowth and systemic inflammation (9, 12, 18). Such disruptions emphasize that post-stroke dysbiosis is not only a taxonomic shift but also a breakdown of microbial functional networks, underscoring the importance of ecological resilience in maintaining host health.

### Limitations

The multivariate models may be overfitted due to the relatively high number of predictors relative to sample size. Penalized regression methods (e.g., Lasso or Ridge regression) may be more appropriate for future analyses. Accordingly, the current multivariate results should be viewed as exploratory.

### Clinical implications

The integration of microbiota profiling, dysbiosis indices, and inflammatory biomarkers offers a multidimensional framework for assessing prognosis in AIS. Our findings suggest that elevated SDI/MDI, combined with biomarker signatures such as high NMDAR, TMAO, iFABP, and LPS with low butyrate and RANKL, could serve as a powerful predictive tool for infection risk and poor functional outcomes. This approach aligns with the emerging concept of the microbiota–gut–brain axis as a determinant of stroke outcomes (1, 3, 12). Moreover, the identification of specific microbial and metabolic signatures opens avenues for therapeutic interventions targeting gut microbiota, such as probiotics, prebiotics, SCFA supplementation, or fecal microbiota transplantation, to restore microbial balance and reduce complications.

This study highlights the critical role of gut microbiota dysbiosis, inflammatory biomarkers, and barrier dysfunction in shaping prognosis after acute ischemic stroke. Reduced microbial diversity, enrichment of pathogenic taxa, depletion of SCFA-producing bacteria, and elevated SDI/MDI scores were strongly associated with systemic infections and adverse biomarker profiles, including elevated NMDAR, TMAO, iFABP, and LPS alongside reduced butyrate and RANKL levels. These findings underscore that post-stroke outcomes are not solely determined by neurological injury but also by complex interactions across the microbiota–gut–brain axis. The integration of microbiome-derived indices with systemic biomarkers offers a promising predictive framework for risk stratification and opens new avenues for therapeutic interventions aimed at restoring microbial balance, strengthening gut barrier integrity, and modulating systemic inflammation. The relatively small sample size (*n* = 80, including 37 infection cases) limits the generalizability and statistical power of the findings. The inclusion of multiple biomarkers, dysbiosis indices, and microbiota taxa increases the risk of model overfitting and false-positive results. Although statistically significant differences were observed (*p* < 0.001 for most biomarkers), corrections for multiple comparisons were not applied, which may overestimate significance. Subgroup analyses (e.g., age, dysphagia) should therefore be regarded as exploratory. Accordingly, the findings of this study should be interpreted as hypothesis-generating and warrant validation in larger, multicenter cohorts with prespecified power analyses. Future studies, particularly prospective clinical trials, are warranted to validate these combined signatures and evaluate microbiota-targeted strategies as adjunctive therapies for improving recovery and reducing complications in acute ischemic stroke patients.

### Translations implications

These findings underscore the potential of microbiota-targeted therapeutic strategies in acute ischemic stroke. Restoration of gut microbial balance through probiotics, prebiotics, or dietary modulation may represent a feasible adjunctive approach to reduce systemic inflammation and infection risk after stroke. Experimental and early clinical studies have shown that supplementation with short-chain fatty acid (SCFA)–producing strains and fiber-rich diets can enhance gut barrier integrity, modulate immune responses, and improve neurological recovery. Future translational efforts integrating microbiome-based diagnostics and therapeutic modulation may help personalize post-stroke care and prevention strategies.

### Limitations

This study has several limitations. The sample size was modest, which may limit statistical power and increase the risk of overfitting in multivariate analyses. Although FDR correction was applied, residual risks of false-positive findings cannot be completely excluded. The cross-sectional nature of the data precludes causal inference. In addition, microbiota composition may be influenced by unmeasured confounders such as diet, medications, or comorbidities. Therefore, these results should be interpreted as hypothesis-generating, requiring validation in larger and longitudinal studies.

### Future directions

Future studies should include larger, multicenter cohorts to validate these findings and minimize model overfitting. Longitudinal follow-up would help determine the temporal dynamics of gut dysbiosis and biomarker fluctuations after stroke. Interventional trials assessing the impact of microbiota-targeted therapies, such as probiotics, SCFA supplementation, or dietary modification, could clarify their potential to improve outcomes and reduce post-stroke complications. Integration of multi-omics approaches—including metagenomics, metabolomics, and immunoprofiling—may also advance precision medicine applications in neurovascular disease.

## Data Availability

The original contributions presented in the study are publicly available. This data can be found here: https://doi.org/10.5281/zenodo.18207270.
